# Phosphotungstic acid (PTA) preferentially binds to collagen- rich regions of porcine carotid arteries and human atherosclerotic plaques observed using contrast enhanced micro-computed tomography (CE-µCT)

**DOI:** 10.3389/fphys.2023.1057394

**Published:** 2023-02-02

**Authors:** A. Hanly, R. D. Johnston, C. Lemass, A. Jose, B. Tornifoglio, C. Lally

**Affiliations:** ^1^ Trinity Centre for Biomedical Engineering, Trinity College Dublin, Dublin, Ireland; ^2^ Department of Mechanical, Manufacturing & Biomedical Engineering, School of Engineering, Trinity College Dublin, Dublin, Ireland; ^3^ Advanced Materials and Bioengineering Research Centre (AMBER), Royal College of Surgeons in Ireland and Trinity College Dublin, Dublin, Ireland

**Keywords:** contrast enhanced micro computed tomography, 3D imaging, cardiac vasculature, atherosclerotic plaques, vascular collagen, phosphotungstic acid

## Abstract

**Background and aims:** Atherosclerotic plaque rupture in the carotid artery can cause small emboli to travel to cerebral arteries, causing blockages and preventing blood flow leading to stroke. Contrast enhanced micro computed tomography (CEμCT) using a novel stain, phosphotungstic acid (PTA) can provide insights into the microstructure of the vessel wall and atherosclerotic plaque, and hence their likelihood to rupture. Furthermore, it has been suggested that collagen content and orientation can be related to mechanical integrity. This study aims to build on existing literature and establish a robust and reproducible staining and imaging technique to non-destructively quantify the collagen content within arteries and plaques as an alternative to routine histology.

**Methods:** Porcine carotid arteries and human atherosclerotic plaques were stained with a concentration of 1% PTA staining solution and imaged using MicroCT to establish the *in situ* architecture of the tissue and measure collagen content. A histological assessment of the collagen content was also performed from picrosirius red (PSR) staining.

**Results:** PTA stained arterial samples highlight the reproducibility of the PTA staining and MicroCT imaging technique used with a quantitative analysis showing a positive correlation between the collagen content measured from CEμCT and histology. Furthermore, collagen-rich areas can be clearly visualised in both the vessel wall and atherosclerotic plaque. 3D reconstruction was also performed showing that different layers of the vessel wall and various atherosclerotic plaque components can be differentiated using Hounsfield Unit (HU) values.

**Conclusion:** The work presented here is unique as it offers a quantitative method of segmenting the vessel wall into its individual components and non-destructively quantifying the collagen content within these tissues, whilst also delivering a visual representation of the fibrous structure using a single contrast agent.

## Graphical Abstract

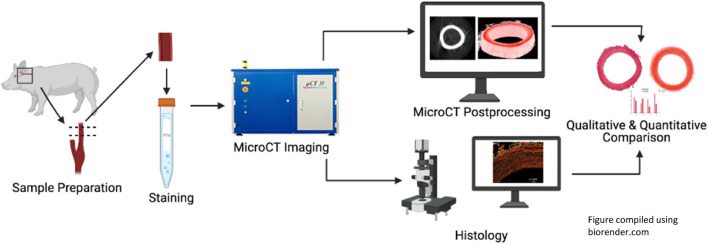

## Introduction

Plaques in the carotid artery can rupture and yield emboli that can migrate, causing blockages in smaller arteries, leading to ischemic stroke (Barnes and Farndale, 1999). Stroke is a leading cause of death and disability in Europe. It is estimated 10% of individuals will die within 30 days of stroke onset and that over 50% of survivors will lose their independence within the first 6 months of recovery (Bamford et al., 1990; Wade, 2012; Wilkins et al., 2017). Prevention remains the best way to reduce mortality and disability as a result of stroke (Ovbiagele and Nguyen-Huynh, 2011). Collagen is known to be the major load bearing component of healthy arterial walls and atherosclerotic plaque tissues, and it is the structure and orientation of collagen within these tissues that dictates their mechanical strength (Fung, 1993; Holzapfel et al., 2000; Johnston et al., 2021). Therefore, understanding the underlying microstructure of the arterial wall and atherosclerotic plaque tissue, along with the mechanisms of rupture, could help to identify vulnerable plaques and prevent plaque rupture. Thus, it is crucial to understand the orientation, density, and coherence of collagen fibres in both healthy and diseased states (Holzapfel and Fratzl, 2008). Current *in-vivo* imaging techniques remain limited in providing a detailed characterization of the vessel and atherosclerotic plaque microstructure due to resolution limitations (Leyssens et al., 2021) and therefore destructive *ex-vivo* techniques are used. Histology remains the gold standard for *in vitro* imaging of collagen in vascular tissue, despite its destructive nature and that it only offers a thin, two-dimensional (2D) snapshot of the tissue (Pagiatakis et al., 2015). Optimised collagen imaging techniques that are inexpensive, fast, and reliable are of great interest, and recent works have attempted to establish new and novel imaging techniques which give a better representation of the 3D vessel microstructure (Nierenberger et al., 2015; Akyildiz et al., 2017; Gaul et al., 2017; Tornifoglio et al., 2020; Robinson et al., 2021; Stone et al., 2021).

Contrast enhanced micro computed tomography (CEµCT, MicroCT) is one such technology that offers the potential to non-destructively image the vessel wall and atherosclerotic plaque at sufficient resolution (Nierenberger et al., 2015; Leyssens et al., 2021; Robinson et al., 2021; Self et al., 2021). MicroCT is an imaging technique which can create highly specific 3D renders, depicting the internal detail of biological samples based on their interaction with X-rays (De Bournonville et al., 2019). Traditionally, MicroCT imaging was reserved for hard, dense tissue such as bone, but over the past decade developments in heavy element staining protocols have established it as a method to image soft tissue (Metscher, 2009a; Metscher, 2009b; Metscher and Müller, 2011; Pauwels et al., 2013). MicroCT imaging offers a unique opportunity to image the tissue microstructure at resolutions similar to histology, with the additional advantage of 3D reconstruction, and without destroying the sample of interest. Furthermore, some papers have reported reversible staining protocols, which can remove the staining solution from the tissue, leaving it entirely unaltered by the CEµCT protocol (Schmidbaur et al., 2015; Self et al., 2021).

Recently, iodine-based stains have been used to image arteries and atherosclerotic plaques with MicroCT, and the resulting images enabled the layers of the arterial wall to be differentiated and the segmentation of calcium deposits (Self et al., 2020; Robinson et al., 2021; Self et al., 2021). However, none of these contrasts provide quantitative insight into the fibrous microstructure of the vessel and atherosclerotic plaque. In 2015, Nierenberger et al. (2015) used a phosphotungstic acid (PTA) staining protocol, initially proposed by Metscher (2009b), to successfully image the microstructure of porcine iliac veins. Their work identified the intima, media, adventitia and connective tissue of the vein, and produced 3D renders which qualitatively showed the orientation of the fibrous structures. Based on studies that report that PTA binds preferentially to collagen (Nemetschek et al., 1979; Karhula et al., 2017; De Bournonville et al., 2019), Nierenberger et al. concluded that it was the collagen microstructure within these vessels which was rendered in 3D. However, the work of Nierenberger et al. did not have histological controls to verify their findings, therefore further exploration of the binding element of PTA to collagen is required. Furthermore, the work of Nierenberger et al. gave no indication of collagen content within their samples and was limited to qualitatively noting the orientation of the microstructure.

Within this study, we tested the hypothesis that the staining protocols presented by Nierenberger et al. (2015) could be adapted and applied to porcine arterial tissue, and to human atherosclerotic plaques. We explored whether, when coupled with MicroCT imaging, these protocols could be used to image the collagen in these tissues in 3D, and yield both qualitative and quantitative information on the collagen content within the tissues. We compared the images obtained from PTA staining and MicroCT imaging to Picrosirius red (PSR) stained histological sections from the same sample to establish if the MicroCT protocols were compatible to routine histology, and to verify if in fact the images were indicative of the underlying collagen microstructure.

## Materials and methods

### Sample preparation

Porcine carotid arteries of 6-month-old white pigs were obtained from an abattoir. Within 3 h the arteries were excised from the connective tissue and frozen at a controlled rate of −1°C/min to −80°C in tissue freezing media made up of 500 mL Gibco RPMI 1640 Medium (21875034, BioSciences), 19.6 g sucrose (S0389, Sigma) and 73.3 mL of the cryoprotectant dimethylsulfoxide (PIER20688, VWR International). The samples were stored at −80°C until ready to image. For imaging, three arteries were thawed at room temperature, rinsed with PBS and any remaining connective tissue was removed using a fresh scalpel. Two 15 mm samples from each of the three vessels were cut (*n* = 6). Each sample was fixed in 5 ml of 10% formalin for 25 h. After fixation they were stored in 70% ethanol until staining.

### Staining

A 1% PTA solution was made up by combining 5 ml of 10% Aqueous PTA (Sigma-Aldrich, HT152) with 45 ml of 70% ethanol. Each of the six samples was submerged in 14 ml of this solution for 16 h. For the final 30 min of staining, the samples were placed on a rotator at 20 RPM. Following staining all samples were dipped in 15 ml of 70% ethanol to remove excess PTA, and then stored in 5 ml of 70% ethanol until scanning.

### MicroCT

Each sample was placed in an 11.5 mm diameter holder and the holder was filled with 70% ethanol such that the sample was submerged. The sample was secured in place using a small piece of low-density polyethylene and the top of the holder was sealed with parafilm. The holder was placed in the MicroCT chamber for imaging. Samples were imaged using a SCANCO Medical AG MicroCT42 with an X-ray energy of 70 kVp and an X-ray intensity of 114 µA. The highest resolution voxel size achievable was 6 µm. The MicroCT scan parameters were based on those of Nierenberger et al. (2015) and are detailed in Supplementary Table S1 of the Supplementary Material S1. After scanning, samples (*n* = 6, from three different vessels) were stored in 70% ethanol and transferred to histological processing.

### De-staining

PTA was removed from the samples (*n* = 6, paired to samples still stained for each vessel) using 0.1 M NaOH, as proposed by Schmidbaur et al. (2015). 0.1 M NaOH was made up by combining 0.2 g of NaOH with 50 ml of DI water. Samples (*n* = 6) were placed in 14 ml of the NaOH solution for 16 h. For the final 30 min of de-staining samples were placed on a rotator set to 20 RPM. Samples were dipped in 15 ml of 70% ethanol to remove excess NaOH and stored in 5 ml of 70% ethanol after de-staining, some were reimaged to prove the efficacy of the washing protocols (*n* = 3) and all six samples were then sent to histological processing.

### Histology

The samples which were stained with PTA and then de-stained (*n* = 6) were step-wise dehydrated in ethanol to xylene, embedded in paraffin wax and sliced at a 6 µm thickness using a microtome (RM-2125RT, Leica). Slices were mounted on slides and stained with Picrosirius Red (PSR). The slides were imaged using an Olympus BX41 microscope with Ocular V2.0 software. Samples were imaged at × 2 and × 10 magnification. Slides were imaged using both Brightfield and Polarised Light Microscopy (PLM). Two PLM images were obtained at 90^o^ to each other and combined to visualise the birefringent collagen and determine the collagen content within the slice.

### Data analysis

The MicroCT data was exported in DICOM image format and the images were analysed using Horos (a free and open-source code software program available from Horosproject.org and sponsored by Nimble Co. LLC d/b/a Purview in Annapolis, MD United States) and OsiriX Lite (an open-source software available from osirix-viewer.com), and MATLAB (MathWorks, Cambridge, UK).

#### Quantitative analysis

The DICOM images were read into MATLAB, and the pixel values within the image were converted to Hounsfield Units using the DICOM header information. Three regions of interest in the internal media and three regions of interest in the external media were manually drawn (as shown in Figure 4B) and the average Hounsfield Unit value in the area was obtained. This was repeated for three slices across each of the samples and averaged.

#### Vessel segmentation

The vessel was segmented into the intima, media and adventitia based on the Hounsfield Units within each of the layers. The thresholds used for each of the layers is included in Supplementary Table S2.

#### Histological analysis

Collagen content was calculated using the established protocol used in Johnston et al. (2021) and is defined as the area of collagen in the combined PLM image divided by the total tissue area in the brightfield image. Specifically, both the 10x PLM image and the corresponding Brightfield image were binarized. The collagen content in the vessel wall was calculated as the number of white pixels in a user specified ROI in the PLM image, divided by the number of white pixels in the same ROI in the brightfield image.

### Application to human atherosclerotic plaques

To establish that this protocol could be adapted for thicker and more heterogeneous plaque tissues, a human plaque obtained from endarterectomy was stained in 15 ml of 1% PTA. The plaque samples were obtained from symptomatic endarterectomy patients at St. James’ hospital, Dublin. Ethical approval for obtaining these plaque specimens in this study was obtained from St. James Hospital ethical committee in compliance with the declaration of Helsinki. Atherosclerotic plaques were washed in phosphate buffered saline and then frozen as described for the porcine carotid tissue above. The plaque was fixed in 15 ml of 10% formalin. After fixation the sample was stored in 70% ethanol until staining. The sample was stained for 68 h in total and the final 30 min of staining was performed on the rotator. The atherosclerotic plaque was scanned using the same scan parameters as the healthy porcine tissue, but due to the large size of the plaque the field of view was increased, such that the highest resolution possible was 8 µm. The images were exported in DICOM format and analysed using Horos.

#### Statistical analysis

Statistical analysis was performed with Prism eight statistical software (GraphPad Software Inc. San Diego, California) *via* a Pearson correlation analysis. This allowed for the relationship between the Hounsfield unit measured from MicroCT to the collagen content observed in histology to be established.

## Results

### PTA staining

**FIGURE 1:**
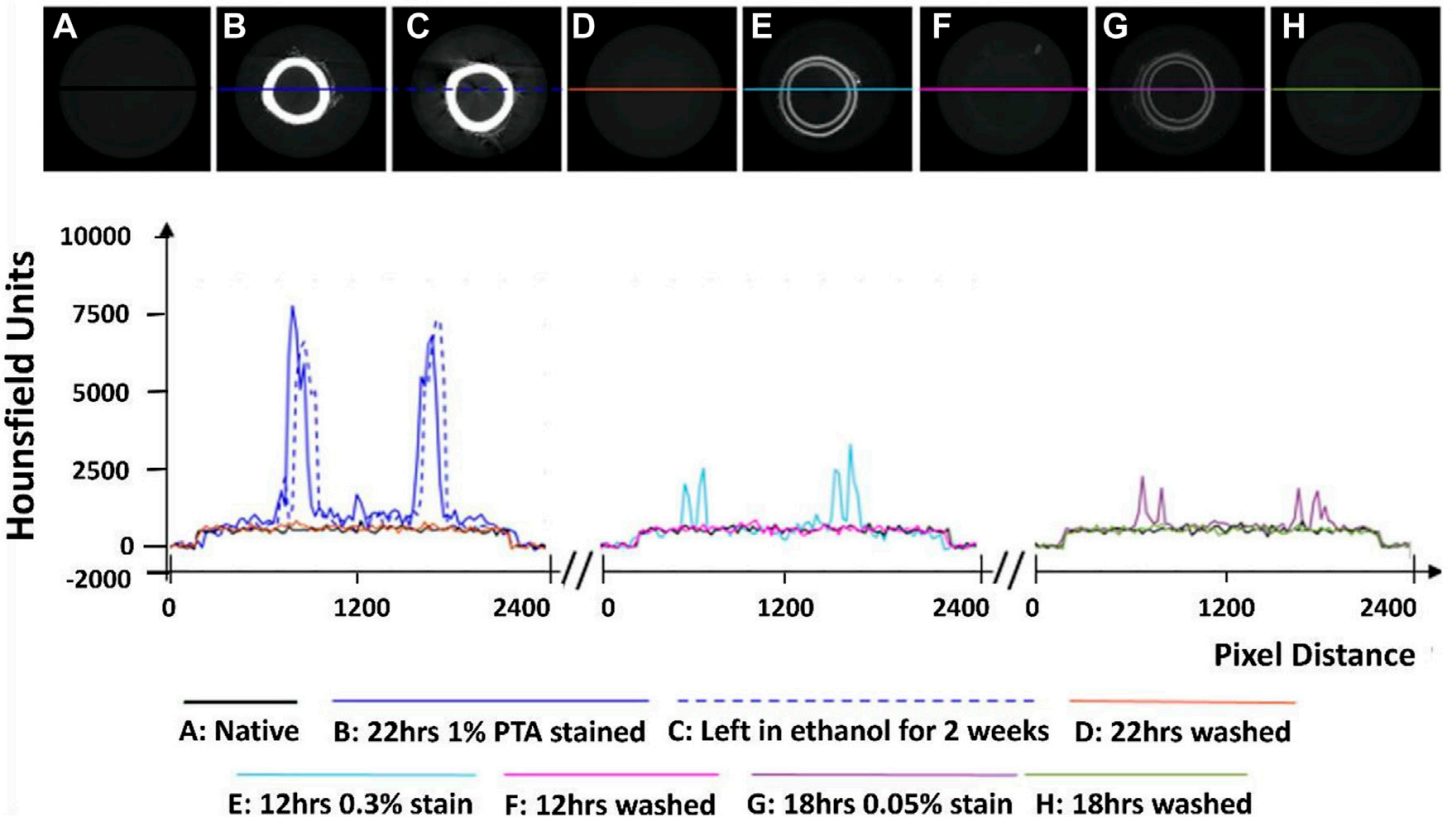
Establishment of the optimum concentration for PTA penetration through arterial tissue samples. **(A)** Native control **(B)** Stained sample for 22 h at 1% PTA **(C)** Preservation in ethanol for 2 weeks **(D)** Removal of stain using NaOH for 22 h **(E)** Stained sample for 12 h at 0.3% PTA **(F)** Removal of stain using NaOH for 12 h. **(G)** Stained sample for 18hrs in 0.05% stain. **(H)** Removal of stain using NaOH for 18 h.

While an unstained artery (termed native) showed no discernible contrast with respect to the ethanol in the MicroCT images (see Figure 1A), PTA-stained arteries could be clearly distinguished from the ethanol background (see Figures 1B, C, E, G).

**FIGURE 2:**
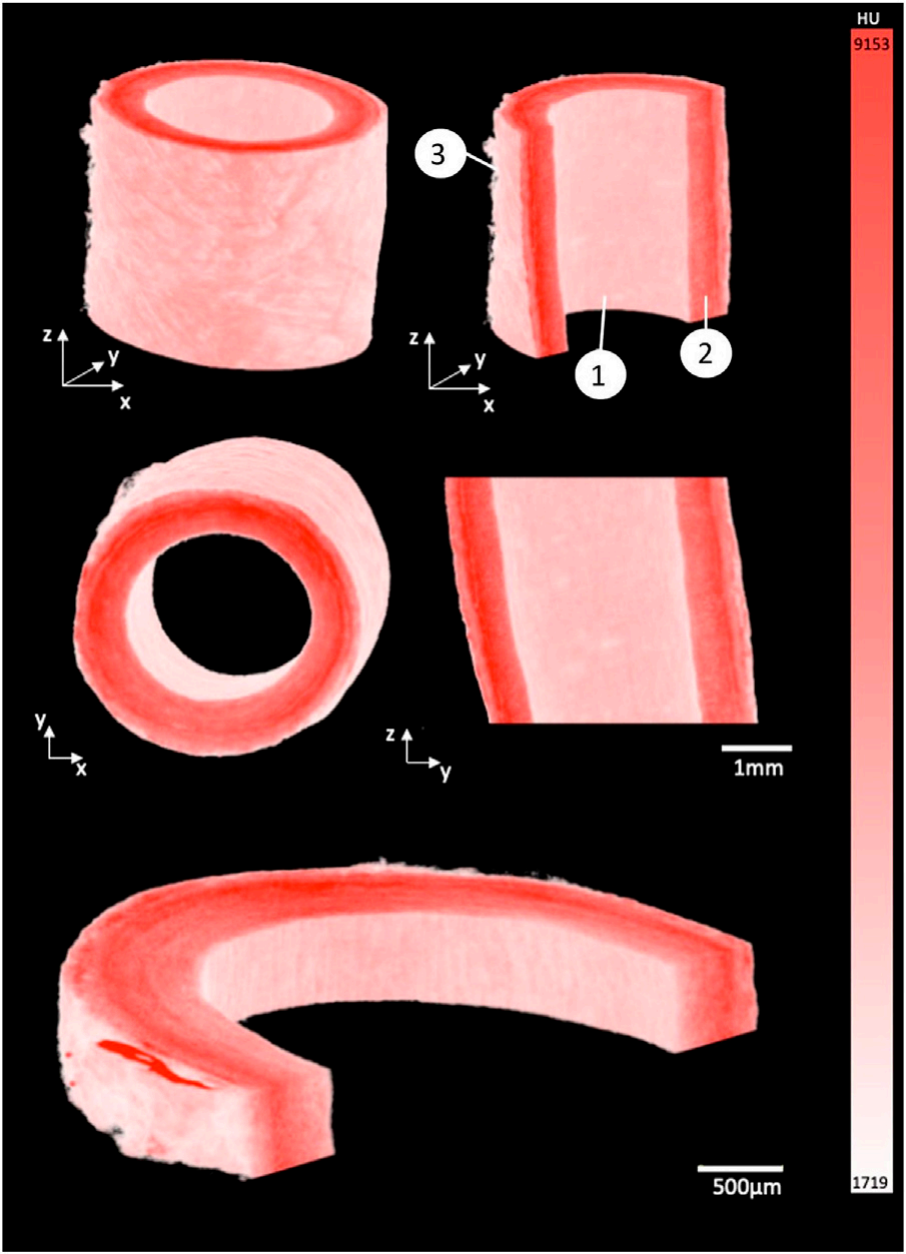
3D render of porcine carotid artery stained for 15 h using 1% PTA. Each image shows different cross-sectional views highlighting the capability to visualize the multiple layers of the vessel wall: (1) Intimal layer, (2) Medial layer, and (3) Adventitial layer.

**FIGURE 3:**
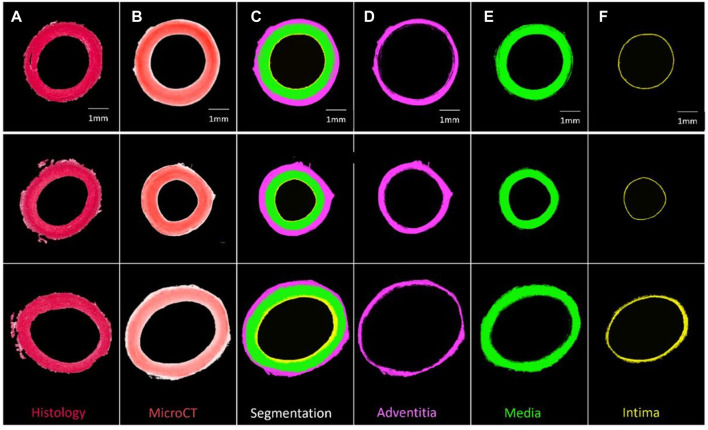
Segmentation of three PTA-stained porcine carotid artery samples into their respective layers **(A)** Histological PSR image, **(B)** PTA stained MicroCT image, **(C)** Segmentation, **(D)** Adventitia, **(E)** Media, and **(F)** Intima.

**FIGURE 4:**
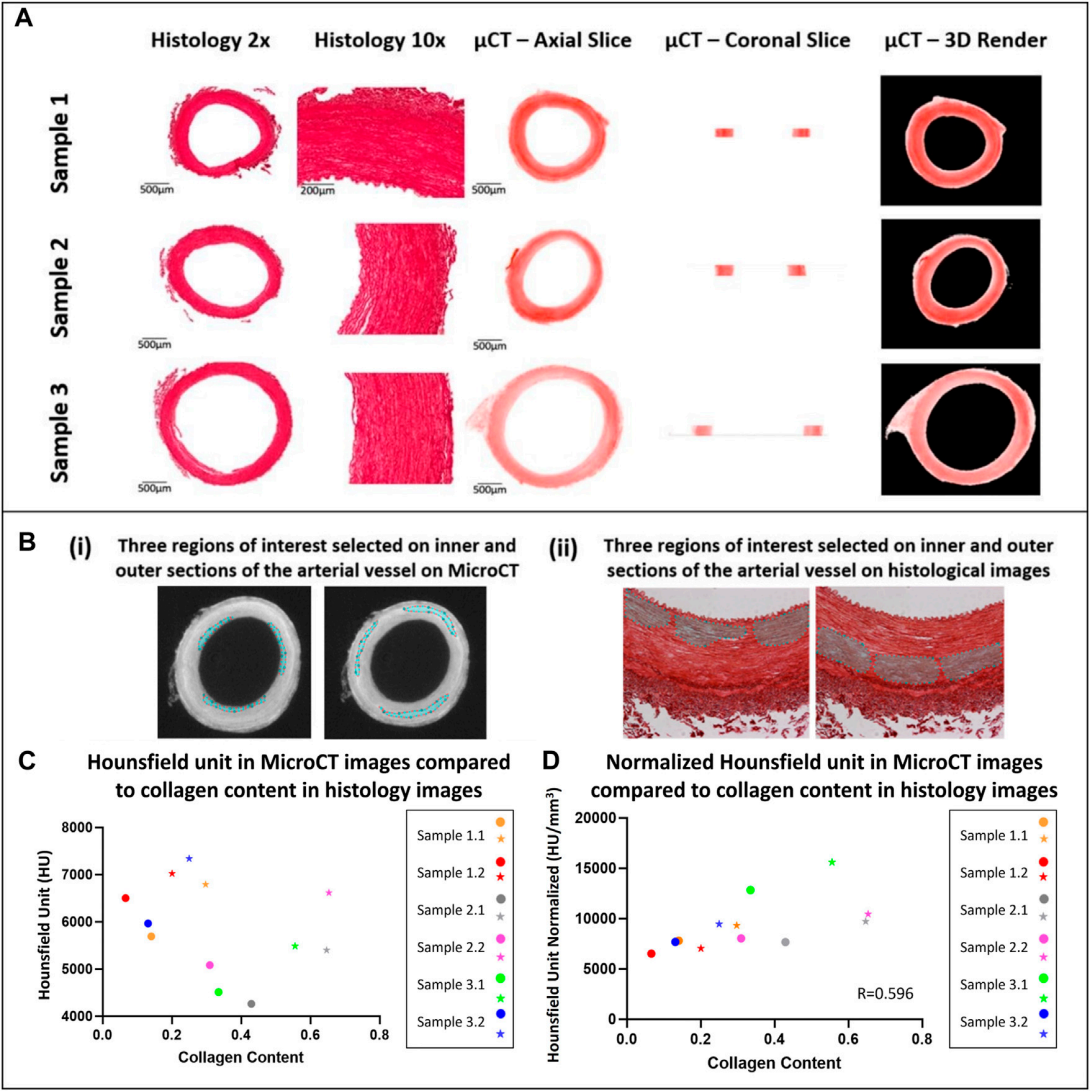
Qualitative and quantitative comparison of MicroCT to histology **(A)** Qualitative comparison observing the overall structure of porcine carotid arteries. **(B)** Region of interest selection for quantitative comparison of the collagen content observed in porcine carotid arteries. **(C)** Increase in collagen content observed in different sections of vessel wall whereby the external media present higher collagen content and HU then the internal media, internal (circles) and external (stars) regions of interest in the media. **(D)** Pearson correlation between HU from MicroCT and collagen content from PS-Red images when normalised with respect to volume.

Initial exploratory work revealed that staining healthy porcine carotid with 0.3% PTA did not allow for complete penetration of the stain through the arterial wall (see Figure 1E), even after 24 h (see Supplementary Figures S1A–C). In contrast, staining a vessel with 1% PTA allowed for full penetration of the stain through the vessel after 22 h (see Figure 1B). Further investigation established the optimal timing for staining was 15 h with 1% PTA concentration and this was used for the quantitative analysis (see Figures 2–4).

Line profiles were generated of representative cross-sections of the native, stained and de-stained arteries (see Figure 1), in order to demonstrate quantitatively the changes in radiopacity. Stronger concentrations of PTA lead to higher radiopacity across the vessel wall and higher pixel intensities, see Figure 1.

### De-staining

Exploratory work also revealed that after storage in 70% ethanol, samples stained with 1% PTA showed no discernible loss of contrast (see Figure 1C). However, when the samples were washed in 0.1 M NaOH, the stain was completely removed from the sample and the pixel profile across the image returned to that of a native vessel (see Figures 1D, F, H). This was shown on all three of the samples which were re-imaged following de-staining.

### 3D render

By using the window level tool in Osirix, a window width of 7884, and a window level of 5211 was set to ensure the samples were easily separated from the background and could be rendered in 3D, see Figure 2. The intima, media and adventitia could be distinguished in the 3D renders, and the fibrous structure within each layer was visible. Along the intima, Voxel intensity appeared to yield contrast parallel to blood flow. In the media a circumferential fibrous alignment could be seen, and the outer layer showed a loose and disorganised fibrous structure.

### Segmentation in 3D space

Samples could be quantitatively segmented into the lumen, intima, media and adventitia, based on the differences in Hounsfield Units for respective regions as outlined in Supplementary Material S1 and Supplementary Table S2. Using these thresholds and the “grow region of interest tool” in Horos, segmentation of individual layers was possible as shown by Figure 3.

### Qualitative comparison of MicroCT to histology

In comparison to conventional histology, MicroCT allowed for planar slices through the samples in the axial, sagittal and coronal planes to be viewed (see Figure 4 for examples), and for the entire artery to be rendered in 3D (see Figure 2). The MicroCT images were not prone to tissue processing defects observed in histology such as wrinkling, tearing or compression. Furthermore, MicroCT images at this resolution showed similar visualisation to 2x histology images (see Figure 4A). Looking at the 2D image slices from our MicroCT and histology datasets, the media and adventitia can clearly be identified, see Figure 4A. In the 3D render of the MicroCT data, the intima, media and adventitial layers of the vessel wall were clearly visible, see Figure 2. A comparison of the images obtained from MicroCT and histology is shown for three representative samples in Figure 4A.

### Quantitative comparison of MicroCT and histology

For each artery, it was observed that regions with higher collagen content had higher Hounsfield Units, see Figure 4C. However, across samples there was no correlation between the Hounsfield Units and the collagen content within the vessel.

It was observed that this was likely due to varying tissue volumes across the samples and the resulting variability in the intensity of the stain across different samples. Thus, the Hounsfield units were normalised with respect to volume. To do this, the volume of each tissue sample imaged was calculated on Osirix and each tissue volume was then divided by the smallest tissue volume to find a normalizing ratio. This ratio was then multiplied by the HU. Once volume was accounted for, the collagen content observed across a number of tissue samples showed a positive moderate correlation with an r = 0.596 between the histology and MicroCT as shown in Figure 4D.

### Application to human atherosclerotic plaques

**FIGURE 5:**
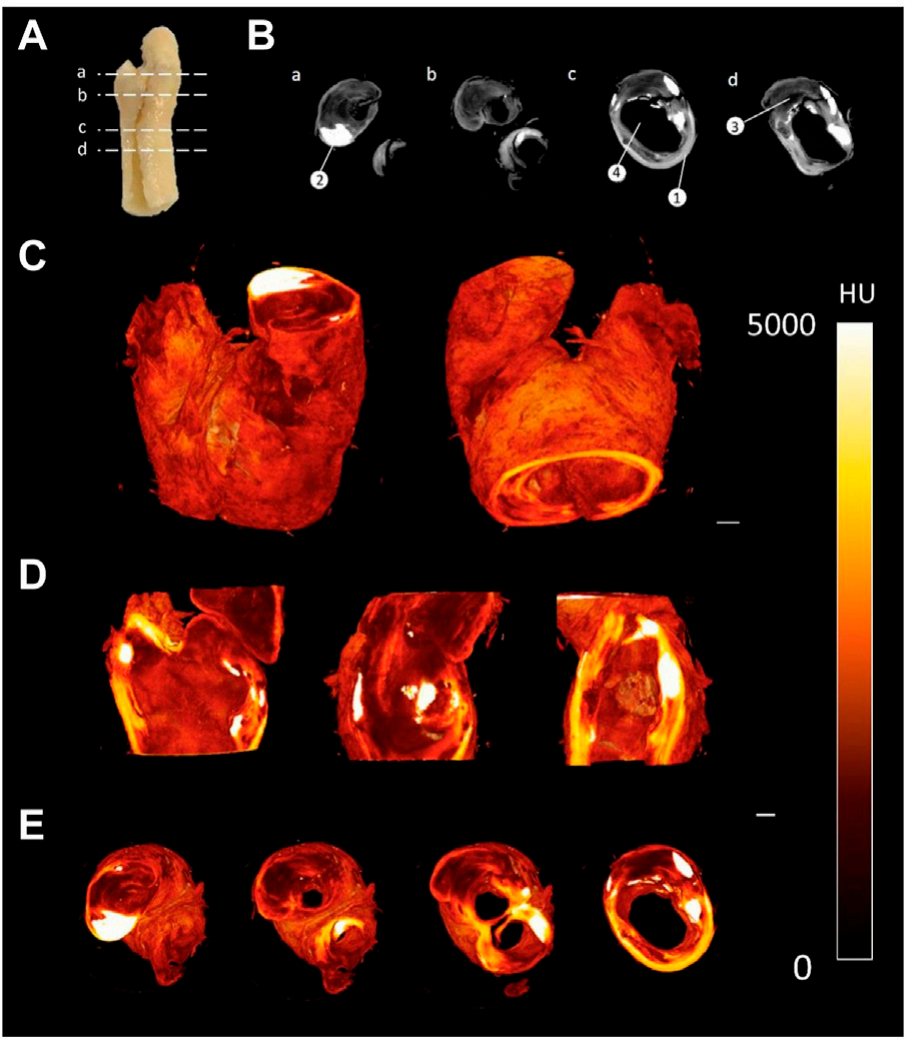
CEµCT of human atherosclerotic plaque **(A)** Slice locations in atherosclerotic plaque. **(B)** Axial cross-sections directly from MicroCT show multiple components within the tissue: (1) Outer wall of plaque, (2) Calcification, (3) Lipid, and (4) Lumen of plaque. **(C)** 3D render of atherosclerotic plaque. **(D)** Sagittal and coronal cross-sections through atherosclerotic plaque. **(E)** Axial cross-sections through atherosclerotic plaque. Scale bar = 1 mm.

The staining protocol was successfully adapted to human atherosclerotic plaques. By leaving the sample in the PTA solution for a longer period of time, the samples could be imaged using the MicroCT and rendered in 3D (see Figure 5). Qualitative assessment revealed distinctive regions within the plaque, including the lumen, lipid rich cores, calcified regions and fibrous caps, as shown in Figure 5B. However, in the atherosclerotic plaque images, the fibrous structure was not as clearly delineated as it was in the images of the healthy arteries.

## Discussion

This data shows that MicroCT imaging, combined with PTA staining, can be used to generate high resolution 3D images of healthy and diseased arterial tissue, and has the capability to differentiate key features of the vessel wall and delineate the fibrous structure within these tissues. While CEµCT has been used to image arterial tissue in the past, the fibrous structures within the vessels were not visible. We found the protocols established by Nierenberger et al. for staining the fibrous structures in veins were not transferrable to porcine arterial tissue, as the 0.3% PTA stain repeatedly failed to penetrate through the vessel wall, even after 24 h. There are a number of plausible reasons for this; firstly, although arteries and veins are composed of the adventitia, media and intima, the wall thickness of veins is significantly thinner than arteries, with larger lumen diameters. Whilst the variation in thickness would affect the staining time, as suggested by Pauwels et al. (2013), the presence of internal and external elastic laminae in arteries could also affect the diffusion capability of PTA through the tissue. Secondly, although veins and arteries are comprised of the same structural layers as mentioned previously, the size of these layers is different. In arterial tissue, the thickest layer is the tunica media which comprises a number of microstructural components such as collagen, elastin and smooth muscle cells. This layer is much thinner in the veins. The relative content of these components in the tissue are different given the different structural and functional requirements of veins and arteries. Using a higher concentration of PTA, we repeatedly saw full penetration of the stain through the vessel wall, provided the samples were left in the stain for 15 h or more. A 1% PTA solution has previously been used by to stain cartilage samples for MicroCT imaging, and a 10% PTA solution has been used to stain mouse plaque samples (Nieminen, et al. 2015; Stadelmann et al., 2021), but to the author’s knowledge this is the first time an established PTA protocol has been used to stain porcine or human arterial tissue with quantitative analysis of the collagen content within these samples.

In the healthy porcine arteries, at an isotropic voxel size of 6 µm the fibrous structure within the vessel wall was visible, and changes in the fibre orientation between the intima, media and adventitia could be visualized. The circumferential fibre alignment seen in the medial layer of the 3D render and the more disorganised fibre alignment in the adventitia agree with what is widely reported in literature to be the fibrous structure of arteries (Gasser et al., 2006; Schriefl et al., 2012; Gaul et al., 2017; Holzapfel and Ogden, 2018). The internal layer of the vessel wall also showed elongated parallel grooves, which corresponds to the parallel alignment of this layer with the blood flow in the vessel (Gasser et al., 2006; Schriefl et al., 2012; Gaul et al., 2017; Holzapfel and Ogden, 2018). Given the 6 µm voxel size used in the current study, the images were not quite as high resolution as those in the Nierenberger et al. study on porcine iliac veins, where the voxel size was 1 µm.

The vessels imaged using MicroCT for this study were also imaged by histology, which further establishes CEµCT as an imaging modality which is complementary to conventional histology. This work has also shown that MicroCT may be an appropriate alternative to histology in some cases. Images from the MicroCT at this resolution are similar to 2x histology images, see Figure 4, and MicroCT imaging presents its own unique advantages over histology. MicroCT is less tedious and faster than conventional histology, preserves tissue integrity and enables rapid 3D visualisation of the tissue. While 3D histopathology methods have been developed, they are slow and intricate, and often rely on a very high number of slides within a z-stack (Roberts et al., 2012; Tornifoglio et al., 2020). The isotropic voxel size for MicroCT imaging means the resolution in the *z*-direction is equal to that in the x and y planes. With MicroCT imaging, cross-sectional images could be obtained in the coronal, sagittal and axial planes at the same resolution, on a single dataset, something that is impossible with histology.

Using Hounsfield units, the layers of the arterial wall could be reproducibly and quantitatively segmented in a manner which shows good agreement with corresponding histology images. Nierenberger et al. (2015) defined layers of the vein wall but based on qualitative assessment and with no histological analysis to confirm their interpretation. Self et al. (2020); Robinson et al. (2021); Self et al. (2021) managed to segment layers of the vessel wall in iodine and iohexol stained plaque tissue, and a similar segmentation has been obtained here for healthy PTA-stained vessels. In fact, this work advances the work of Robinson et al. (2021), because here the boundary of every layer of the arterial wall was defined quantitatively, whereas in the work of Robinson et al. the media and the intima were manually segmented. In the work of Self et al. (2020) it is stated that the vessel layers were segmented based on the changes in grey levels across the vessel wall, but the authors provide no further detail which could be used for comparison to the methods here. They do however state their method is “subjective in nature”. Thus, the method of segmentation provided in this paper offers a significant improvement where provided the stain concentration and imaging parameters are kept constant, the method can be objectively and repeatably applied to multiple vessels.

Samples which were washed in 0.1 M NaOH lost the contrast generated by PTA and had pixel profiles like that of unstained arteries (Figure 1). It was necessary to use a specialized washing solution because little loss of contrast was observed in samples stored in 70% ethanol for 2 weeks, indicating the stain remains stable in the sample over time, which has been reported previously (Metscher, 2009b; Nierenberger et al., 2015). The washing protocols build on the work of Schmidbaur et al. (2015). who showed 0.3% PTA could be washed from preserved Syllis Armillaris using 0.1 M NaOH. In their study the PTA stain was removed from two out of three 0.3 mm samples after 6 hours. In this study, stain removal was achieved in all three re-imaged vessels, in which the wall thickness ranged from 0.8—1.2 mm. By matching both the wash volume and wash time to the staining volume and time, the 1% PTA was removed.

Quantitative analysis showed a moderate positive correlation between the MicroCT Hounsfield unit and measured collagen content from histology as shown by Figure 4C, with an r value of 0.596 once volume was accounted for. However, it was also shown that within blood vessels, the PTA concentration was higher in the more collagen rich external media than the internal media, suggesting that PTA is binding preferentially to collagen and is visualised in the MicroCT. Furthermore, it is shown that higher collagen content is observed in the medial layer of the arterial wall when compared to intima and adventitia and this is also expected as its observed in the literature (Camasão and Mantovani, 2021). This indicates that CEµCT combined with PTA staining could be used to identify more collagen rich regions within samples. To establish a more quantitative metric and compare the collagen content across multiple samples, a normalising ratio with respect to volume would be required either for the staining solution or during post-processing.

Using the amended protocol for PTA staining of atherosclerotic plaque tissue, several tissue components could be clearly visualised and quantified using MicroCT. However, detail on the microstructural level is not the same as observed in the porcine arterial vessels due to a different resolution being used. The reason for this lower resolution is due to the larger FOV size that is required. As the voxel size for imaging the plaque was 8µm, it was unlikely that individual collagen fibres would be observed. Nonetheless it has been shown that the staining protocols are transferrable to human plaque tissue and improved image resolution will be further explored in future work. Furthermore, another worthwhile investment would be to validate the segmentation and quantitative analysis by performing a region-specific analysis with the aid of identifiable landmarks and compare between the MicroCT and histology on the atherosclerotic plaque tissue.

Overall, MicroCT offers significant advantages over conventional histological processing to observe collagen content and is a feasible tool for *ex-vivo* analysis of arterial tissue and atherosclerotic plaques. Firstly, it gives comparable representation of the collagen content in arterial vessels to the gold standard, namely histology. Secondly, it allows for imaging the entire sample in 3D, offering the capability to view the out of plane information (*z*-direction) that is important in establishing the overall structural integrity of the tissue (Johnston et al., 2021). Thirdly, preservation of the sample allows for mechanical testing to be performed to further understand the mechanics (Brunet et al., 2021). These samples are difficult to obtain and it is important to harness as much information as possible, especially when it comes to human tissue. Lastly, MicroCT is much faster to obtain and not destructive when compared to histology and can act as a validation to other *in-vivo* imaging modalities such as MRI that strive to establish specificity to collagen and microstructural arrangement (Tornifoglio et al., 2020; Stone et al., 2021).

A number of limitations can be noted in this study, firstly, the selectivity of the binding of PTA to collagen has long been debated in literature (Silverman and Glick, 1969; Nemetschek et al., 1979). Collagen degradation has a significant effect on the binding of PTA in articular cartilage tissue suggesting its specificity of binding to collagen (Karhula et al., 2017); however, this has not been established in arterial tissue. While this research indicates PTA binds more intensely to collagen rich areas, future work should work to further establish the specificity of the stain. Furthermore, MicroCT is a non-destructive imaging technique that preserves the mechanical properties of the vessel; however, no mechanical testing has been performed in this study. A focus for future research will be to investigate the staining of fresh arterial samples that allow for mechanical tests to be performed, similar to Brunet et al. (2021). Lastly, due to resolution constraints, the maximum resolution that could be achieved was 6 μm. Given that collagen fibres are approximately the same size as the resolution set and that collagen fibre diameters can vary throughout the thickness of the vessel wall (Ushiki, 2002; Chow et al., 2014; Niestrawska et al., 2022), the resolution is insufficient to fully delineate fibres and perform fibre tracking to establish fibre orientation similar to diffusion tensor imaging protocols (Flamini et al., 2010; Tornifoglio et al., 2020). Future work will look at building a customized rig to hold the sample and that is compatible with the MicroCT system that enables translation closer to the x-ray beam so a smaller field of view and subsequently a better resolution can be obtained. Lastly, although the ultimate goal would be to perform these studies *in-vivo,* further development of contrast agents specific to microstructural components that are biocompatible and non-toxic is required and an area of further research.

## Conclusion

This work supports the use of MicroCT imaging combined with PTA staining as a novel and robust protocol for assessing the structure and collagen content of healthy arterial tissue and has established that the methodology is adaptable to human atherosclerotic plaque. We have built on the findings of other key studies in the field. With one singular stain, we have achieved similar results in arteries that Nierenberger et al. (2015) obtained by imaging the fibrous structure of venous walls, demonstrated a substantial improvement on the work of Robinson et al. (2021), who based on histology segmented multiple layers of the vessel wall using iodine and MicroCT, and confirmed the results of Self et al. (2021) whereby we have shown that the stain can be washed from the arterial tissue samples after scanning. Furthermore, this study is first to use MicroCT imaging to analyse the collagen content in arterial tissue and to include a quantitative analysis of CEµCT imaging of blood vessels. Characterising the collagen content in intact vessels in 3D will improve our understanding of the role of collagen in the progression of atherosclerotic diseases, which in turn may lead to better clinical indicators of plaque stenosis.

## Data Availability

The raw data supporting the conclusion of this article will be made available by the authors, without undue reservation.

## References

[B1] Akyildiz A. C. Chai C. K. Oomens C. W. J. van der Lugt A. Baaijens F. P. T. Strijkers G. J. (2017). 3D fiber orientation in atherosclerotic carotid plaques. J. Struct. Biol. 200, 28–35. 10.1016/j.jsb.2017.08.003 28838817

[B2] Bamford J. Dennis M. Sandercock P. Warlow C. Burn J. (1990). The frequency, causes and timing of death within 30 days of a first stroke: The Oxfordshire Community Stroke Project. J. Neurol. Neurosurg. Psychiatry. 53, 824–829. 10.1136/jnnp.53.10.824 2266360 PMC488240

[B3] Barnes M. J. Farndale R. W. (1999). Collagens and atherosclerosis. Exp. Gerontol. 34, 513–525. 10.1016/S0531-5565(99)00038-8 10817807

[B4] Brunet J. Pierrat B. Adrien J. Maire E. Curt N. Badel P. (2021). A novel method for *in vitro* 3D imaging of dissecting pressurized arterial segments using X-ray microtomography. Exp. Mech. 61, 147–157. 10.1007/s11340-020-00645-x

[B5] Camasão D. B. Mantovani D. (2021). The mechanical characterization of blood vessels and their substitutes in the continuous quest for physiological-relevant performances. A critical review. Mat. Today Bio. 10, 100106. 10.1016/j.mtbio.2021.100106 PMC8050780 33889837

[B6] Chow M. J. Turcotte R. Lin C. P. Zhang Y. (2014). Arterial extracellular matrix: A mechanobiological study of the contributions and interactions of elastin and collagen. Biophys. J. 106, 2684–2692. 10.1016/j.bpj.2014.05.014 24940786 PMC4070071

[B7] De Bournonville S. Vangrunderbeeck S. Kerckhofs G. (2019). Contrast-enhanced microCT for virtual 3D anatomical pathology of biological tissues: A literature review. Contrast Media Mol. Imaging 2019, 8617406. 10.1155/2019/8617406 30944550 PMC6421764

[B8] Flamini V. Kerskens C. Moerman K. M. Simms C. K. Lally C. (2010). Imaging arterial fibres using diffusion tensor imaging-feasibility study and preliminary results. EURASIP J. Adv. Signal Process. 2010, 904091–904098. 10.1155/2010/904091

[B9] Fung Y. C. (1993). Biomechanics: Mechanical properties of living tissue. 2nd edn. New York: Springer-Verlag.

[B10] Gasser T. C. Ogden R. W. Holzapfel G. A. (2006). Hyperelastic modelling of arterial layers with distributed collagen fibre orientations. J. R. Soc. Interface. 3, 15–35. 10.1098/rsif.2005.0073 16849214 PMC1618483

[B11] Gaul R. T. Nolan D. R. Lally C. (2017). Collagen fibre characterisation in arterial tissue under load using SALS. J. Mech. Behav. Biomed. Mat. 75, 359–368. 10.1016/j.jmbbm.2017.07.036 28787646

[B12] Holzapfel G. A. Gasser T. C. Ogden R. W. (2000). A new constitutive framework for arterial wall mechanics and a comparative study of material models. J. Elast. 61, 1–48. 10.1023/A:1010835316564

[B13] Holzapfel G. A. Ogden R. W. (2018). Biomechanical relevance of the microstructure in artery walls with a focus on passive and active components. Am. J. Physiol. - Hear. Circ. Physiol. 315, H540–H549. 10.1152/ajpheart.00117.2018 29799274

[B14] Holzapfel G. (2008). “Collagen in arterial walls: Biomechanical aspects,” in Collagen structure and mechanics, collagen struct. Mech. Editor Fratzl P. (Heidelberg, Germany: Springer V).

[B15] Johnston R. D. Gaul R. T. Lally C. (2021). An investigation into the critical role of fibre orientation in the ultimate tensile strength and stiffness of human carotid plaque caps. Acta Biomater. 124, 291–300. 10.1016/j.actbio.2021.02.008 33571712

[B16] Karhula S. S. Finnilä M. A. Lammi M. J. Ylärinne J. H. Kauppinen S. Rieppo L. (2017). Effects of articular cartilage constituents on phosphotungstic acid enhanced micro-computed tomography. PLoS One 12, 01710755–e171112. 10.1371/journal.pone.0171075 PMC5279764 28135331

[B17] Leyssens L. Pestiaux C. Kerckhofs G. (2021). A review of *ex vivo* x-ray microfocus computed tomography-based characterization of the cardiovascular system. Int. J. Mol. Sci. 22, 3263. 10.3390/ijms22063263 33806852 PMC8004599

[B18] Metscher B. D. (2009). Micro CT for comparative morphology: Simple staining methods allow high-contrast 3D imaging of diverse non-mineralized animal tissues. BMC Physiol. 9, 11. 10.1186/1472-6793-9-11 19545439 PMC2717911

[B19] Metscher B. D. (2009). MicroCT for developmental biology: A versatile tool for high-contrast 3D imaging at histological resolutions. Dev. Dyn. 238, 632–640. 10.1002/dvdy.21857 19235724

[B20] Metscher B. D. Müller G. B. (2011). MicroCT for molecular imaging: Quantitative visualization of complete three-dimensional distributions of gene products in embryonic limbs. Dev. Dyn. 240, 2301–2308. 10.1002/dvdy.22733 21901786

[B21] Nemetschek T. Riedl H. Jonak R. (1979). Topochemistry of the binding of phosphotungstic acid to collagen. J. Mol. Biol. 133, 67–83. 10.1016/0022-2836(79)90251-1 529283

[B40] Nieminen (2015). Determining collagen distribution in articular cartilage using contrast-enhanced micro-computed tomography. 23 9, 1613–1621. 10.1016/j.joca.2015.05.004 PMC4565718 26003951

[B22] Nierenberger M. Rémond Y. Ahzi S. Choquet P. (2015). Assessing the three-dimensional collagen network in soft tissues using contrast agents and high resolution micro-CT: Application to porcine iliac veins. Comptes Rendus - Biol. 338, 425–433. 10.1016/j.crvi.2015.04.009 26033495

[B23] Niestrawska J. A. Pukaluk A. Babu A. R. Holzapfel G. A. (2022). Differences in collagen fiber diameter and waviness between healthy and aneurysmal abdominal aortas. Microsc. Microanal. 1–15. 10.1017/S1431927622000629 35545876

[B24] Ovbiagele B. Nguyen-Huynh M. N. (2011). Stroke epidemiology: Advancing our understanding of disease mechanism and therapy. Neurotherapeutics 8, 319–329. 10.1007/s13311-011-0053-1 21691873 PMC3250269

[B25] Pagiatakis C. Galaz R. Tardif J. C. Mongrain R. (2015). A comparison between the principal stress direction and collagen fiber orientation in coronary atherosclerotic plaque fibrous caps. Med. Biol. Eng. Comput. 53, 545–555. 10.1007/s11517-015-1257-z 25752768

[B26] Pauwels E. Van Loo D. Cornillie P. Brabant L. Van Hoorebeke L. (2013). An exploratory study of contrast agents for soft tissue visualization by means of high resolution X-ray computed tomography imaging. J. Microsc. 250, 21–31. 10.1111/jmi.12013 23432572

[B27] Roberts N. Magee D. Song Y. Brabazon K. Shires M. Crellin D. (2012). Toward routine use of 3D histopathology as a research tool. Am. J. Pathol. 180, 1835–1842. 10.1016/j.ajpath.2012.01.033 22490922 PMC3538002

[B28] Robinson S. T. Levey R. E. Beatty R. Connolly D. Dolan E. B. Osborne N. H. (2021). A versatile technique for high-resolution three-dimensional imaging of human arterial segments using microcomputed tomography. JVS Vasc. Sci. 2, 13–19. 10.1016/j.jvssci.2020.08.001 34617054 PMC8489243

[B29] Schmidbaur H. Keklikoglou K. Metscher B. D. Faulwetter S. (2015). Exploring methods to remove iodine and phosphotungstic acid stains from zoological specimens. Bruker MicroCT User Mtg Abstr. 21, 116–123. Available at: https://www.semanticscholar.org/paper/Exploring-methods-to-remove-iodine-and-acid-stains-Schmidbaur-Keklikoglou/9f9375488adcd3be6766a492b1977b0c68372e2d

[B30] Schriefl A. J. Zeindlinger G. Pierce D. M. Regitnig P. Holzapfel G. A. (2012). Determination of the layer-specific distributed collagen fibre orientations in human thoracic and abdominal aortas and common iliac arteries. J. R. Soc. Interface. 9, 1275–1286. 10.1098/rsif.2011.0727 22171063 PMC3350738

[B31] Self T. S. Ginn-hedman A. Kaulfus C. N. Newell-fugate A. E. Weeks B. R. Heaps C. L. (2020). Iodine-enhanced micro-computed tomography of atherosclerotic plaque morphology complements conventional histology. Atherosclerosis 313, 43–49. 10.1016/j.atherosclerosis.2020.09.012 33022583 PMC7655693

[B32] Self T. S. Ginn-Hedman A. M. Newell-Fugate A. E. Weeks B. R. Heaps C. L. (2021). Iodine-based contrast staining improves micro-computed tomography of atherosclerotic coronary arteries. MethodsX 8, 101297. 10.1016/j.mex.2021.101297 34434817 PMC8374297

[B33] Silverman L. Glick D. (1969). The reactivity and staining of tissue proteins with phosphotungstic acid. J. Cell Biol. 40, 761–767. 10.1083/jcb.40.3.761 4179634 PMC2107646

[B34] Stadelmann V. A. Boyd G. Guillot M. Bienvenu J. G. Glaus C. Varela A. (2021). Automatic quantification of atherosclerosis in contrast-enhanced MicroCT scans of mouse aortas *ex vivo* . Int. J. Biomed. Imaging. 2021, 4998786. 10.1155/2021/4998786 34594369 PMC8478544

[B35] Stone A. J. Tornifoglio B. Johnston R. D. Shmueli K. Kerskens C. Lally C. (2021). Quantitative susceptibility mapping of carotid arterial tissue *ex vivo*: Assessing sensitivity to vessel microstructural composition. Magn. Reson. Med. 86 (5), 2512–2527. 34270122 10.1002/mrm.28893

[B36] Tornifoglio B. Stone A. J. Johnston R. D. Shahid S. S. Kerskens C. Lally C. (2020). Diffusion tensor imaging and arterial tissue: Establishing the influence of arterial tissue microstructure on fractional anisotropy, mean diffusivity and tractography. Sci. Rep. 10, 20718–20812. 10.1038/s41598-020-77675-x 33244026 PMC7693170

[B37] Ushiki T. (2002). Collagen fibers, reticular fibers and elastic fibers. A comprehensive understanding from a morphological viewpoint. Arch. Histol. Cytol. 65, 109–126. 10.1679/aohc.65.109 12164335

[B38] Wade D. T. (2012). Impact commentaries. Functional abilities after stroke: Measurement, natural history and prognosis. J. Neurol. Neurosurg. Psychiatry. 83, 770. 10.1136/jnnp-2011-301689 22228728

[B39] Wilkins E. Wilson L. Wickramasinghe K. Bhatnagar P. Leal J. Luengo-Fernandez R. (2017). European cardiovascular disease statistics 2017, European heart network, brussels. Eur. Cardiovasc. Dis. Stat. 34, 3028–3034.

